# Supplementary material for: Pyruvate Dehydrogenase Kinase 1 inhibition mediated oxidative phosphorylation enhancement in cartilage promotes osteoarthritis progression
Source: BMC Musculoskelet Disord. 2023 Jul 20;24:597. doi: 10.1186/s12891-023-06585-6 (PMC10357736; doi:10.1186/s12891-023-06585-6)
Supplement: Supplementary file 1 — Additional file 1. [file 12891_2023_6585_MOESM1_ESM.zip › BP.Gsea.1653623667859/GOBP_REGULATION_OF_ASTROCYTE_DIFFERENTIATION.html]

Details for gene set GOBP\_REGULATION\_OF\_ASTROCYTE\_DIFFERENTIATION[GSEA]

|  || Dataset | 55457\_collapsed\_to\_symbols.cls.cls #OA\_versus\_normal.cls.cls #OA\_versus\_normal\_repos |
| Phenotype | cls.cls#OA\_versus\_normal\_repos |
| Upregulated in class | normal |
| GeneSet | GOBP\_REGULATION\_OF\_ASTROCYTE\_DIFFERENTIATION |
| Enrichment Score (ES) | -0.63958144 |
| Normalized Enrichment Score (NES) | -2.0666168 |
| Nominal p-value | 0.0017605633 |
| FDR q-value | 0.016629744 |
| FWER p-Value | 0.115 |
Table: GSEA Results Summary

  

Fig 1: Enrichment plot: GOBP\_REGULATION\_OF\_ASTROCYTE\_DIFFERENTIATION      
 Profile of the Running ES Score & Positions of GeneSet Members on the Rank Ordered List

  

| SYMBOL | TITLE | RANK IN GENE LIST | RANK METRIC SCORE | RUNNING ES | CORE ENRICHMENT || 1 | TREM2 | triggering receptor expressed on myeloid cells 2 [Source:HGNC Symbol;Acc:HGNC:17761] | 342 | 0.726 | 0.0361 | No |
| 2 | F2 | "coagulation factor II, thrombin [Source:HGNC Symbol;Acc:HGNC:3535]" | 4745 | 0.092 | -0.2979 | No |
| 3 | SERPINE2 | serpin family E member 2 [Source:HGNC Symbol;Acc:HGNC:8951] | 4873 | 0.082 | -0.3007 | No |
| 4 | NR1D1 | nuclear receptor subfamily 1 group D member 1 [Source:HGNC Symbol;Acc:HGNC:7962] | 6974 | -0.078 | -0.4571 | No |
| 5 | NR2E1 | nuclear receptor subfamily 2 group E member 1 [Source:HGNC Symbol;Acc:HGNC:7973] | 7660 | -0.132 | -0.4989 | No |
| 6 | ID4 | "inhibitor of DNA binding 4, HLH protein [Source:HGNC Symbol;Acc:HGNC:5363]" | 8174 | -0.169 | -0.5242 | No |
| 7 | NF1 | neurofibromin 1 [Source:HGNC Symbol;Acc:HGNC:7765] | 9194 | -0.247 | -0.5820 | No |
| 8 | MAG | myelin associated glycoprotein [Source:HGNC Symbol;Acc:HGNC:6783] | 9490 | -0.273 | -0.5814 | No |
| 9 | GPR37L1 | G protein-coupled receptor 37 like 1 [Source:HGNC Symbol;Acc:HGNC:14923] | 9519 | -0.276 | -0.5598 | No |
| 10 | BIN1 | bridging integrator 1 [Source:HGNC Symbol;Acc:HGNC:1052] | 10123 | -0.333 | -0.5778 | No |
| 11 | BMP2 | bone morphogenetic protein 2 [Source:HGNC Symbol;Acc:HGNC:1069] | 10919 | -0.420 | -0.6034 | Yes |
| 12 | KDM4A | lysine demethylase 4A [Source:HGNC Symbol;Acc:HGNC:22978] | 10936 | -0.423 | -0.5681 | Yes |
| 13 | PRPF19 | pre-mRNA processing factor 19 [Source:HGNC Symbol;Acc:HGNC:17896] | 11269 | -0.467 | -0.5536 | Yes |
| 14 | DAB1 | DAB adaptor protein 1 [Source:HGNC Symbol;Acc:HGNC:2661] | 11815 | -0.552 | -0.5483 | Yes |
| 15 | ID2 | inhibitor of DNA binding 2 [Source:HGNC Symbol;Acc:HGNC:5361] | 11852 | -0.560 | -0.5028 | Yes |
| 16 | CNTN2 | contactin 2 [Source:HGNC Symbol;Acc:HGNC:2172] | 11858 | -0.561 | -0.4548 | Yes |
| 17 | EPHA4 | EPH receptor A4 [Source:HGNC Symbol;Acc:HGNC:3388] | 11863 | -0.563 | -0.4066 | Yes |
| 18 | LDLR | low density lipoprotein receptor [Source:HGNC Symbol;Acc:HGNC:6547] | 12009 | -0.595 | -0.3665 | Yes |
| 19 | LIF | LIF interleukin 6 family cytokine [Source:HGNC Symbol;Acc:HGNC:6596] | 12085 | -0.615 | -0.3193 | Yes |
| 20 | NTRK3 | neurotrophic receptor tyrosine kinase 3 [Source:HGNC Symbol;Acc:HGNC:8033] | 12157 | -0.632 | -0.2703 | Yes |
| 21 | CLCF1 | cardiotrophin like cytokine factor 1 [Source:HGNC Symbol;Acc:HGNC:17412] | 12360 | -0.695 | -0.2260 | Yes |
| 22 | IL6 | interleukin 6 [Source:HGNC Symbol;Acc:HGNC:6018] | 12460 | -0.730 | -0.1708 | Yes |
| 23 | IL6ST | interleukin 6 cytokine family signal transducer [Source:HGNC Symbol;Acc:HGNC:6021] | 12505 | -0.749 | -0.1096 | Yes |
| 24 | NOTCH1 | notch receptor 1 [Source:HGNC Symbol;Acc:HGNC:7881] | 12555 | -0.776 | -0.0465 | Yes |
| 25 | HES1 | hes family bHLH transcription factor 1 [Source:HGNC Symbol;Acc:HGNC:5192] | 12676 | -0.849 | 0.0174 | Yes |
Table: GSEA details [plain text format]

  

Fig 2: GOBP\_REGULATION\_OF\_ASTROCYTE\_DIFFERENTIATION      
 Blue-Pink O' Gram in the Space of the Analyzed GeneSet

  

Fig 3: GOBP\_REGULATION\_OF\_ASTROCYTE\_DIFFERENTIATION: Random ES distribution      
 Gene set null distribution of ES for **GOBP\_REGULATION\_OF\_ASTROCYTE\_DIFFERENTIATION**
